# microRNA-99a acts as a tumor suppressor and is down-regulated in bladder cancer

**DOI:** 10.1186/1471-2490-14-50

**Published:** 2014-06-23

**Authors:** Yougang Feng, Yongming Kang, Yue He, Jun Liu, Bo Liang, Ping Yang, Zhou Yu

**Affiliations:** 1 Department of Urology, Suining Central Hospital, 127 Deshengxi Road, Suining, Chuanshan District 629000, P.R. China

**Keywords:** Bladder cancer, miR-99a, Circulation miRNA

## Abstract

**Background:**

Increasing evidences have documented that microRNAs (miRNAs) act as oncogenes or tumor suppressors in a variety types of cancer. The discovery of tumor associated miRNAs in serum of patients gives rise to extensive investigation of circulating miRNAs in many human cancers which support the use of plasma/serum miRNAs as noninvasive means of cancer detection. However, the aberrant expression of miRNAs and the circulating miRNAs in bladder cancer are less reported.

**Methods:**

We used Taqman probe stem-loop real-time PCR to accurately measure the levels of miR-99a in bladder cancer cell lines, 100 pairs of bladder cancer tissues, the adjacent non-neoplastic tissues and plasma collected from bladder cancer patients or control patients. miR-99a mimics were re-introduced into bladder cancer cells to investigate its role on regulating cell proliferation which was measured by CCK-8 assay and cell cycle analysis.

**Results:**

miR-99a was significantly down-regulated in bladder cancer tissues, and even the lower expression of miR-99a was correlative with the more aggressive phenotypes of bladder cancer. Meanwhile, enforced expression of miR-99a can inhibit the cell proliferation of bladder cancer cells. Furthermore, investigation of the expression of miR-99a in plasma of bladder cancer patients showed that miR-99a was also decreased in plasma of bladder cancer patients. The results strongly supported miR-99a as the potential diagnostic marker of bladder cancer.

**Conclusions:**

Our data indicated that miR-99a might act as a tumor suppressor in bladder cancer and was significantly down-regulated in development of bladder cancer.

## Background

Bladder cancer is one of the most frequent malignancies in the world. The most common type of bladder cancer is urothelial carcinoma of the bladder. Chromosomal anomalies, genetic polymorphisms, genetic and epigenetic alterations have been reported to be included in the tumorigenesis and progression of bladder cancer [1]. Many molecules involved in these alterations may serve as diagnostic markers of tumor growth and disease progression. Currently, many protein-coding genes and specific genomic regions are the most popular used molecular markers of bladder cancer, such as members of the RAS family, differentially methylated DNA locus [2-4].

MicroRNAs (miRNAs) are endogenous small non-coding RNAs that play crucial roles in multiple biological processes through regulating mRNAs for cleavage or translational repression. Recent studies have documented that miRNAs acted as oncogenes or tumor suppressors in a variety types of cancer, such as lung, breast, hepatic, and pancreatic cancer [5-10]. Several innate properties of miRNAs make them attractive as potential biomarkers. MiRNAs can be detected easily in small amount samples using specific and sensitive quantitative real-time PCR; miRNAs are stable against degradation and can be detectable in bodily fluids including serum, plasma, saliva, urine and tears [11,12]. Furthermore, expression profiles of miRNAs would be changed in the plasma and/or serum of cancer patients and miRNAs have been shown to be released from tumor cells to the circulation [13]. So circulating miRNAs will be a novel class of non-invasive biomarkers for cancer diagnosis and prognosis. However, the expression profile of miRNAs in bladder cancer and miRNAs which may serve as diagnostic markers of bladder cancer are less reported compared with the abroad reports in other cancer types.

miR-99a was proved to be down-regulated in bladder cancer patients by deep sequencing in nine bladder urothelial carcinoma patients [14], low-grade bladder cancer patients [15] and was also reported to act as a tumor suppressor in several other cancer types. For example, miR-99a could promote apoptosis by targeting mTOR in human esophageal squamous cell carcinoma [16]. In addition, miR-99a induces G1-phase cell cycle arrest and suppresses tumorigenicity in renal cell carcinoma [17]. Down-regulation of miR-99a in oral squamous cell carcinomas also contributes to the growth and survival of oral cancer cells [18]. However, the expression pattern of miR-99a in large numbers of bladder cancer patients and its roles in bladder cancer are unknown.

In this study, we used Taqman probe stem-loop real-time PCR to accurately measure the levels of miR-99a in 100 pairs of bladder cancer tissues and the adjacent non-neoplastic tissues to exclude the differences of miR-99a expression in different individuals. We found that miR-99a was significantly down-regulated in bladder cancer tissues and enforced expression of miR-99a repressed the proliferation of bladder cancer cells. Furthermore, investigation of the expression of miR-99a in the plasma of bladder cancer patients showed that miR-99a was also decreased in plasma of bladder cancer patients which strongly supported miR-99a as the potential diagnostic marker of bladder cancer.

## Methods

### Cell culture and transfections

The human bladder cancer cell lines (J82, HT1376, RT4, T24 and TCCSUP) and immortalized human bladder epithelium (HCV29 and HU609) cells were propagated in DMEM (Invitrogen) supplemented with 10% FCS at 37°C in 5% CO_2_ cell culture incubator. miR-99a mimics and scramble control mimics were obtained from Dharmacon (Austin, TX, USA) and transfected with DharmFECT1 (Dharmacon, Austin, TX, USA) in HT1376 and J82 cells at a final concentration of 50 nM.

### Patients and specimens

The human clinical samples were collected from surgical specimens from 100 patients with bladder cancer at Suining Central Hospital. The corresponding adjacent non-neoplastic tissues from the macroscopic tumor margin were isolated at the same time and used as controls. All samples were immediately snapped frozen in liquid nitrogen and stored at -80°C until RNA extraction.

Whole blood samples were prospectively collected from bladder cancer patients and control patients without urologic malignancies. Whole blood (5–8 ml) was collected in an ethylene diamine tetracetic acid (EDTA) tube. The sample was centrifuged twice at 4°C. Plasma (supernatant after second centrifugation) was then stored at -80°C. The Clinical Research Ethics Committee of Suining Central Hospital approved the research protocols and written informed consent was obtained from the participants.

### RNA extraction, cDNA synthesis, and real-time PCR assays

Total RNA was extracted from tissues and cells using Trizol reagent (Invitrogen, CA, USA) according to the manufacturer’s instructions. Total RNA of plasma was isolated using a commercially available kit (mirVana; miRNA Isolation Kit, Applied Biosystems, Carlsbad, CA) according to the manufacturer’s protocol. RNA was quantified and cDNA was synthesized by M-MLV reverse transcriptase (Invitrogen) from 2 μg of total RNA. A stem-loop RT primer was used for the reverse transcription. Quantitative RT-PCR was performed in a Bio-Rad CFX96 real-time PCR System (Bio-Rad, CA, USA) using TaqMan probes (Applied Biosystems, Foster City, CA, USA) according to the manufacturer’ s instructions. The PCR conditions were as follows: 95°C for 30 s, followed by 40 cycles of 95°C for 5 s and 60°C for 34 s. The data were normalized using the endogenous U6 snRNA. The 2-ΔΔCT method was used in the analysis of PCR data. Primer sequences are presented in Table 1.

**Table 1:** Sequence of primers used in qRT-PCR

**Primer**	**Sequence(5′ → 3′)**
miR-99a-RT	GTCGTATCCAGTGCAGGGTCCGAGGTATTCGCACTGGATACGACCACAAGA
miR-99a-forward	GCTGGAGAACCCGTAGATCCGAT
miR-99a-reverse	GTGCAGGGTCCGAGGT
miR-99a-probe	FAM-ATACGACCACAAGATCGG-MGB
U6-RT	AAAATATGGAACGCTTCACGAATTTG
U6-forward	CTCGCTTCGGCAGCACATATACT
U6-reverse	ACGCTTCACGAATTTGCGTGTC
U6-probe	FAM-CCATGCTAATCTTCTCTGTA-MGB

### Cell proliferation assay

To measure the effect of miRNA mimics on cell proliferation, cells were incubated in 10% CCK-8 (DOJINDO) diluted in culture media at 37°C until visual color conversion appeared. Proliferation rates were determined at 12, 24, 48, 72, 96 h post-transfection, and quantification was done on a microtiter plate reader (Spectra Rainbow, Tecan) according to the manufacturer’s protocol.

### Cell cycle analysis

HT1376 and J82 cells were harvested and washed once at 4°C in PBS containing 0.5% BSA then added ice cold 70% ethanol. The fixed cells were immediately stored at -20°C for at least 24 hours. Cells were washed twice in ice cold PBS to remove ethanol and then resuspended in PBS with 25 μg/ml RNase A and 50 μg/ml Propidium Iodide at 37°C for 1 hour. Flow cytometry was performed using a Beckman Coulter and analyzed with ModFit.

### Statistics

The statistical analyses for miR-99a expression in clinical samples, correlation of miR-99a expression with patients’ clinicopathological variables were conducted using the Bonferroni multiple-comparison test. The other statistical analyses were evaluated by Independent samples *T* test (two-tailed). P ≤ 0.05 was considered statistically significant.

## Results

### miR-99a is down-regulated in bladder cancer cells

**Figure 1:**
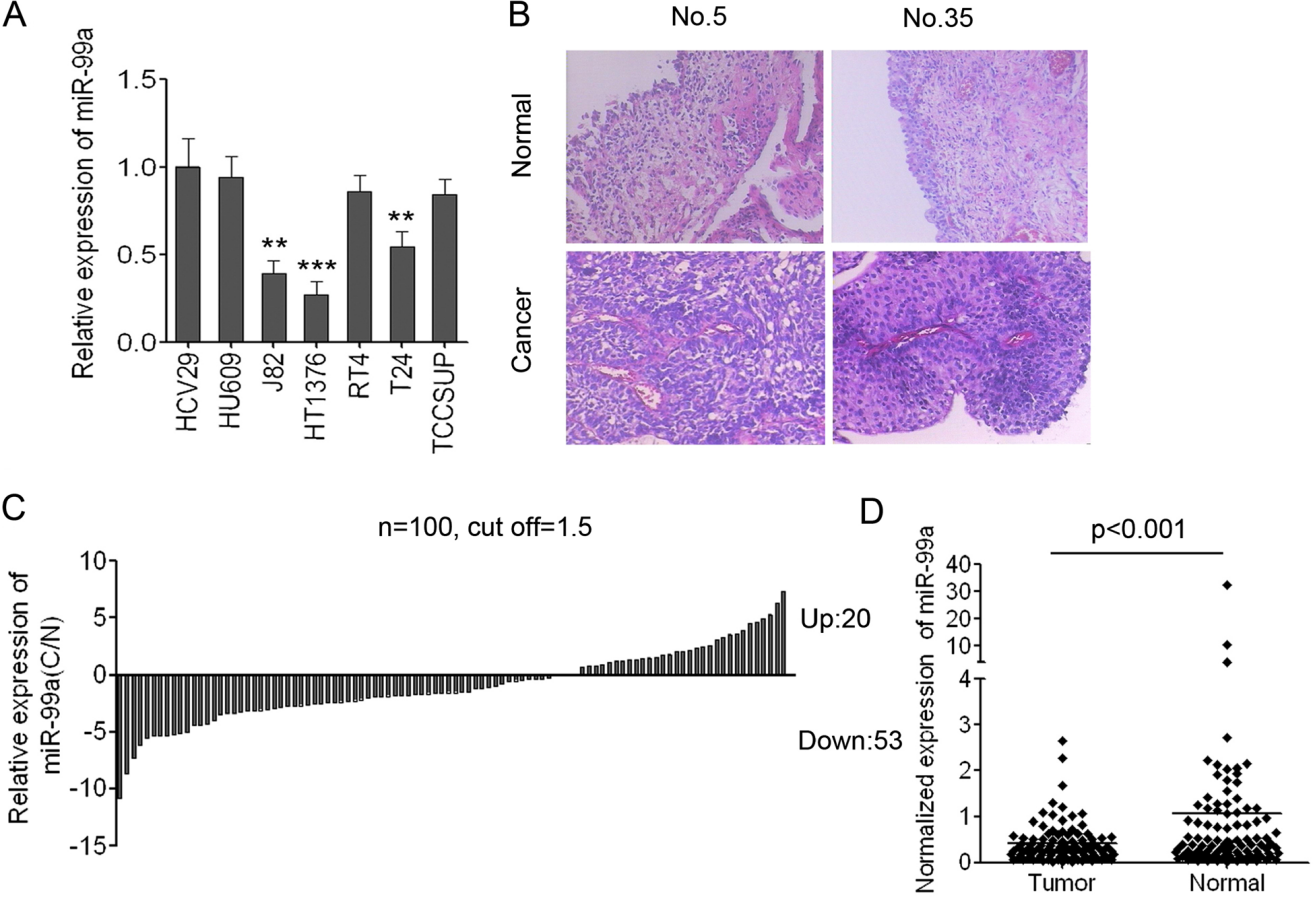
**miR-99a is significantly down-regulated in bladder cancer cell lines and in bladder cancer tissues. (A)** The expression level of miR-99a in two immortalized human bladder epithelium cells (HCV29 and HU609) and five bladder cancer cell lines (J82, HT1376, RT4, T24 and TCCSUP). Data are shown as mean ± s.d. (n = 3); **indicates P-value < 0.01; ***indicates P-value < 0.001. **(B)** HE staining of the bladder cancer **(C)** tissue and adjacent non-neoplastic tissues (N) of 2 patients with bladder cancer. **(C)** The relative expression of miR-99a in 100 pairs of bladder cancer **(C)** and adjacent non-neoplastic tissues (N). **(D)** Normalized expression of miR-99a in 100 pairs of bladder cancer and adjacent normal tissues.

To analyze the expression of miR-99a in bladder cancer, q-PCR using Taqman probes was conducted to measure the levels of miR-99a. We firstly examined the expression of mature miR-99a in five human bladder cancer cell lines (J82, HT1376, RT4, T24 and TCCSUP) and immortalized human bladder epithelium (HCV29 and HU609) cells. The expression level of miR-99a in HU609 and HCV29 was significantly higher than that in the three bladder cancer cell lines (T24, HT1376, and J82) and was non-significantly but observably higher than the levels in the other two bladder cancer cell lines (TCCSUP and RT4) (Figure 1A). These data demonstrated that the down-regulation of miR-99a might be relevant to the genesis and development of bladder cancer.

### miR-99a is down-regulated in bladder cancer tissues compared with the corresponding adjacent non- neoplastic tissues

To further analyze the expression of miR-99a in patients with bladder cancer, we measured the levels of miR-99a in 100 pairs of bladder cancer tissues (C) and the adjacent non-neoplastic tissues (N). Figure 1B showed the representative HE staining of the bladder cancer (C) tissue and adjacent non-neoplastic tissues (N) of 2 patients with bladder cancer. The results of PCR showed that 53/100 (53%) of cases had reduced levels of miR-99a in bladder cancer tissues compared with the corresponding non-neoplastic tissues when the cutoff was set up as 1.5 (Figure 1C). There were 20/100 (20%) of cases which had increased levels of miR-99a in bladder cancer tissues compared with the adjacent non-neoplastic tissues, 27/100 (27%) of cases in whom the expression of miR-99a was unchanged in bladder cancer tissues when the cutoff was set up as 1.5. The results also showed that the average expression of miR-99a in bladder cancer samples was significantly lower than that in the adjacent non-neoplastic tissues (p < 0.001) (Figure 1D). Collectively, the data indicated that miR-99a was significantly attenuated in tumor tissues compared with adjacent normal tissues and might act as a tumor suppressor in bladder cancer.

### Low-level expression of miR-99a is associated with aggressive phenotypes of bladder cancer

**Figure 2:**
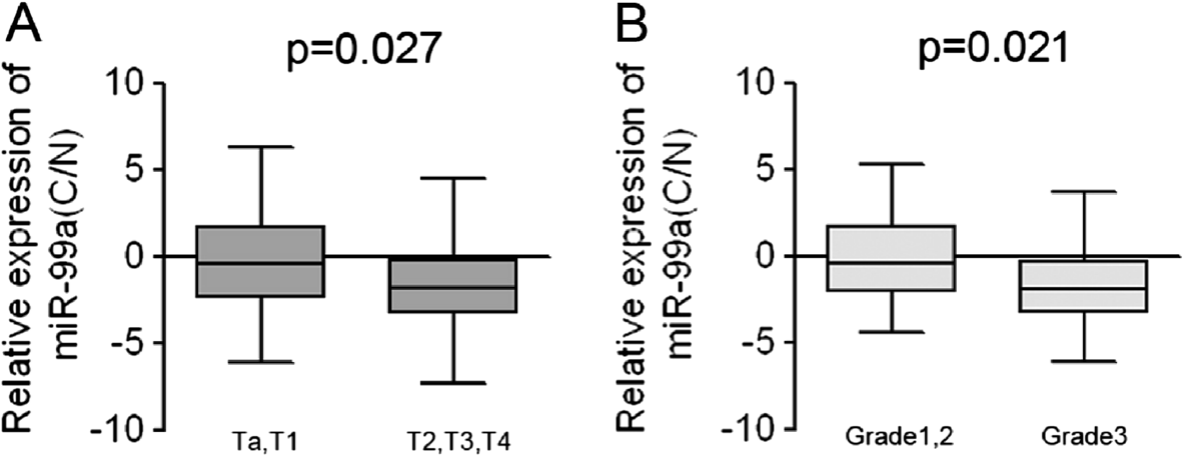
**Correlation of the relative expression of miR-99a with the clinicopathological characteristics of patients with bladder cancer. (A)** The correlation of miR-99a expression with tumor stages of bladder cancer tissues. **(B)** The correlation of miR-99a expression with tumor grades of bladder cancer tissues.

To further investigate the correlation between the expression of miR-99a and the clinicopathological characteristics, the relative expression of miR-99a in 100 pairs of bladder cancer tissues and adjacent normal tissues was statistically analyzed. The clinicopathological features of bladder cancer patients were summarized in Table 2. Correlation analysis showed that low-level expression of miR-99a in bladder cancer was significantly associated with a more extensive muscle invasion (*p* < 0.05, stage Ta, 1 vs. stage T2, 3, 4) (Figure 2A) and a more aggressive tumor phenotype (*p* < 0.05, grade 1, 2 vs. grade 3) (Figure 2B). The data also demonstrated that the expression level of miR-99a had no correlation with age, gender and histological type. The correlation of lower levels of miR-99a with the more aggressive phenotype of bladder cancer strongly indicated that miR-99a played important roles in bladder carcinogenesis as a tumor suppressor.

**Table 2:** Clinicopathological features of bladder cancer patients

**Variables**	**Patients, n**	
	**Total**	**Lower miR-99a**
	**(n = 100)**	**(n = 53)**
**Histology**		
TCC	83	35
TCC with aberrant differentiation	17	18
**Gender**		
Male	75	38
Female	25	15
**Age**		
≥60	62	34
<60	38	19
**Stage**		
Ta	34	15
T1	25	10
T2	18	11
T3	13	9
T4	10	8
**Grade**		
1	25	7
2	40	18
3	35	28
**Smoker**		
Current	30	15
Ex	44	21
Never	26	17
**Recurrence**		
Yes	47	21
No	53	32
**Progression**		
Yes	33	21
No	67	32

### Enforced expression of miR-99a suppresses bladder cancer cell growth

**Figure 3:**
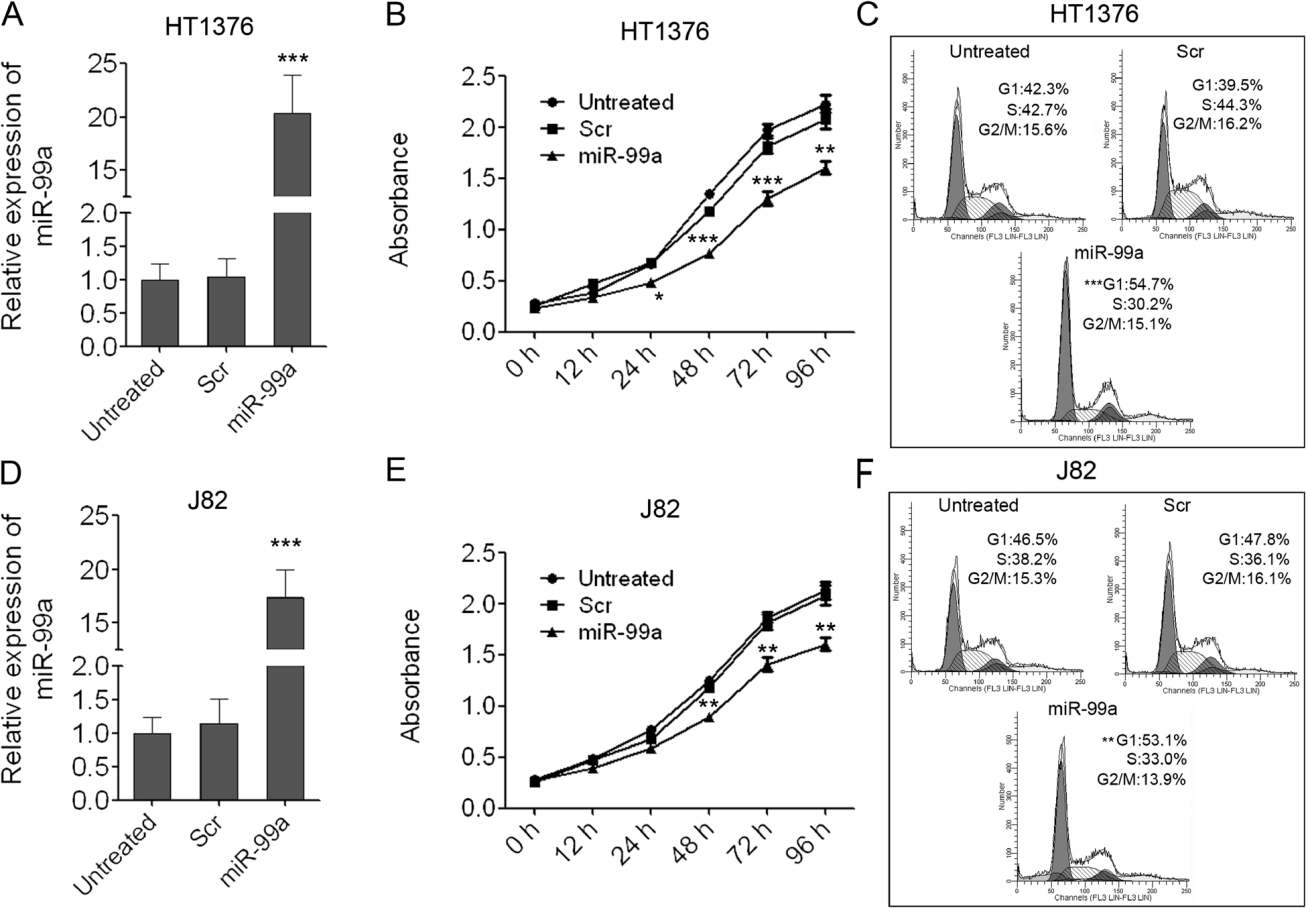
**Enforced expression of miR-99a inhibits cell proliferation of bladder cancer cells. (A)** Overexpression of miR-99a in HT1376 cells was confirmed by qRT-PCR. **(B)** The cell growth of HT1376 at 0, 12, 24, 48, 72, 96 hours post transfection which was detected by CCK-8 assay. **(C)** Cell cycle distribution of HT1376 cells transfected with miR-99a mimics. **(D)** Overexpression of miR-99a in J82 cells was confirmed by qRT-PCR. **(E)** The cell growth of J82 at 0, 12, 24, 48, 72, 96 hours post transfection which was detected by CCK-8 assay. **(F)** Cell cycle distribution of J82 cells transfected with miR-99a mimics. Data are shown as mean ± s.d. (n = 3); **indicates P-value < 0.01; ***indicates P-value < 0.001.

To explore the role of miR-99a in bladder carcinogenesis, we overexpressed miR-99a in the two bladder cancer cell lines HT1376 and J82 in which expression of miR-99a was lower than the other bladder cancer cell lines. Successful overexpression of miR-99a upon transfection in the two bladder cancer cell lines was confirmed by q-PCR. As shown in Figure 3A and 3D, miR-99a was overexpressed about 20 folds and 18 folds than the scramble control or untreated cells in HT1376 and J82 cells respectively. As demonstrated by CCK-8 growth assays at 0, 12, 24, 48, 72, 96 hours after mimic transfection, overexpression of miR-99a reduced cell proliferation in both the two cell lines, whereas the scramble control had no effect on cell proliferation compared with the untreated cells (Figure 3B, E). The suppression of miR-99a on cell proliferation of HT1376 cells was a little stronger than that on J82 cells. The results improved that miR-99a acted as a tumor suppressor in bladder cancer and the down-regulation of miR-99a in bladder tissues would lead to the unlimited cell proliferation. We also checked the cell cycle distribution in HT1376 and J82 cells transfected with miR-99a mimics. The results showed that miR-99a could increase the fraction of cells in G1 phases in HT1376 and J82 cells which suggested that miR-99a might inhibit bladder cancer cell growth through arresting the G1/S transition (Figure 3C, 3F).

### miR-99a is also down-regulated in the plasma of patients with bladder cancer

**Figure 4:**
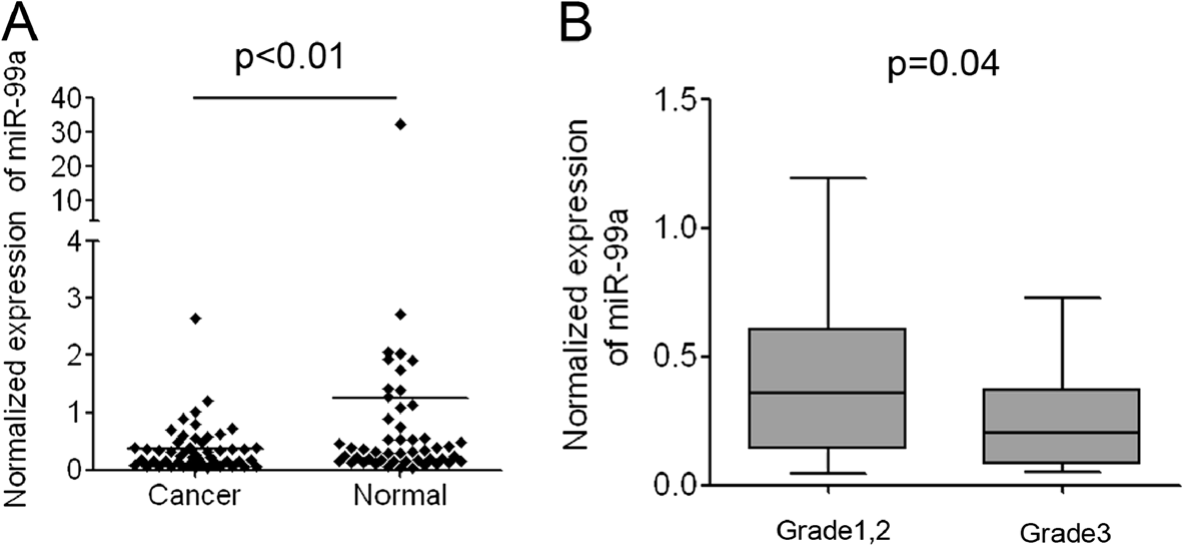
**Expression of miR-99a in the plasma of patients with bladder cancer. (A)** Normalized expression of miR-99a in the plasma of 50 patients with bladder cancer and 50 healthy individuals. **(B)** Correlation of miR-99a expression in the plasma with the tumor grades of bladder cancer.

To explore the diagnostic potential of miR-99a in bladder cancer, we detected the expression of miR-99a in the plasma of 50 patients with bladder cancer and 50 healthy individuals. The data demonstrated that the average level of miR-99a in the bladder cancer patients was significantly lower than that in the healthy individuals (Figure 4A). The expression profile of miR-99a in the plasma was consistent with its down-regulation in bladder cancer tissues. The results suggested that miR-99a could be released from the bladder epithelium to blood and down-regulation of miR-99a in plasma might origin from decreased expression of miR-99a in cancer tissues. The low-level expression of miR-99a in the plasma of bladder cancer patients was also significantly associated with a more aggressive tumor phenotype (*p* < 0.05, grade 1, 2 vs. grade 3, Figure 4B). The decreased expression of miR-99a in the plasma of bladder cancer patients suggested that miR-99a could be developed to a potential diagnostic marker which can be combined with other miRNAs’ expression to detect bladder cancer.

## Discussion

The aberrant expression of miRNAs in bladder cancer has been studied in recent years. Some miRNAs have been reported to be up-regulated in bladder cancer tissues. For example, miR-129 was the most commonly up-regulated and its up-regulation was associated with poor outcome [19]; the expression of miR-96 and miR-183 in urine was significantly correlated with tumor stage and grade, and their expressions were significantly decreased after radical surgery [20]; miR-133b and miR-518c were also strongly up-regulated in bladder cancer tissues [19]. Meanwhile, some miRNAs were reported to be down-regulated in cancer tissues and might function as tumor suppressors. miR-200 family members were lower in urine sediment of bladder cancer patients and increased significantly following surgery which suggested this microRNA family could be used as diagnostic and prognostic markers of bladder cancer [21]. miR-92 and miR-33 were reported to be down-regulated in the plasma of patients with bladder cancer and the expression of these two miRNAs was inversely correlated with the clinical stage of the cancer [22]. Because miRNAs are small, easy to deliver, stable against degradation and easy to be detected, these aberrant expression miRNAs in bladder cancer are attractive as potential biomarkers and new targets for bladder cancer therapy. However, the potential diagnostic and therapeutic roles of these miRNAs in great numbers of clinical samples are just at the beginning and need to be explored further.

Among the miRNAs which were aberrantly expressed in bladder cancer, miR-99a was reported to be down-regulated in bladder cancer patients by deep sequencing in nine bladder urothelial carcinoma patients [14]. Catto et al. also reported the expression of miR-99a was down-regulated in low-grade bladder cancer patients and also identified a target of miR-99a in bladder cancer progression [15]. Different to these reports, we investigated miR-99a expression in tumor tissues and the adjacent non-neoplastic tissues which were derived from a common patient instead of different individuals to exclude the differences of miR-99a expression in different individuals. In addition, we detected miR-99a expression in more clinical samples and also in the plasma of bladder cancer patients. Our investigation of miR-99a levels in 100 pairs of bladder cancer tissues and adjacent normal tissues showed that miR-99a was authentically decreased in bladder cancer tissues. Moreover, the lower level of miR-99a was correlative with more aggressive phenotype of bladder cancer. Reintroduction of miR-99a into the bladder cancer cells which had lower expression of miR-99a could inhibit the unlimited cell proliferation. Collectively, the data indicated that miR-99a functioned as a tumor suppressor in bladder cancer. The roles of miR-99a in regulating bladder cancer cell migration, invasion and apoptosis and the detailed mechanism will be our further directions.

Earlier studies discovered that extracellular miRNAs circulated in the bloodstream and the circulating miRNAs were remarkably stable. Detection of elevated levels of tumor associated miRNAs in serum of patients with diffuse large B-cell lymphoma [23] leads to wide investigation of circulating miRNAs in many human cancers, including breast cancer [24], lung cancer [25], prostate cancer [26], and renal cell carcinoma [27] and so on. The expression profile of miRNAs in serum/plasma of the patients with bladder cancer was also investigated and some important circulating miRNAs in bladder cancer had been identified [28,29]. These studies support the use of serum/plasma miRNAs as noninvasive means of bladder cancer detection. However, there are rare reports about miRNAs down-regulated in the plasma of patients with bladder cancer relative to the extensive studies of miRNAs up-regulated in plasma. We found that the levels of miR-99a in the plasma of bladder cancer was decreased which was consistent with its low level in the cancer tissues although it was unknown how the down-regulation of miR-99a in a relatively small number of tumor cells can affect the plasma miR-99a levels. We think there are two possible explanations for that: 1) The down-regulation of miR-99a in the cancer tissue was significant enough to be able to affect plasma miR-99a levels, meanwhile, the Taqman probe stem-loop real-time PCR was sensitive enough to detect the faint change of miR-99a levels in plasma. 2) The down-regulation of miR-99a in the plasma not only origin from the tumor cells but also from the immunocytes in the tumor microenvironment which needs to be improved further. The decreased expression of miR-99a in the plasma and cancer tissues of bladder cancer patients supported that miR-99a can be developed as a new diagnostic marker for bladder cancer detection.

## Conclusion

In summary, we determined the low expression of miR-99a in the cancer tissues and plasma of patients with bladder cancer and also indicated the tumor suppressor role of miR-99a in bladder cancer. Our data provided the potential diagnostic and therapeutic roles of miR-99a in bladder cancer.

## Competing interests

The authors declare that they have no competing interests.

## Authors’ contributions

Y-MK conceived the project; Y-GF designed the experiments and carried out the majority of the experiments; YH,BL and JL helped to collect clinical samples. PY and ZY helped to culture cells; all authors discussed the results; Y-MK and Y-GF wrote the manuscript. All authors read and approved the final manuscript.

## Pre-publication history

The pre-publication history for this paper can be accessed here:

http://www.biomedcentral.com/1471-2490/14/50/prepub
